# Editorial: Neuromuscular and kinematic dynamics in human movement adaptation

**DOI:** 10.3389/fnhum.2025.1724225

**Published:** 2025-11-11

**Authors:** Rajat Emanuel Singh, Jennifer L. Davies, Catherine Purcell

**Affiliations:** 1 Department of Kinesiology at Northwestern College, Orange City, IA, United States; 2 School of Healthcare Sciences, Cardiff University, Wales, United Kingdom

**Keywords:** locomotion adaptation, human development, injury, neurological disorder, cerebral palsy, stroke, ACL, Parkinson's disease

## Introduction

Our neuromuscular system exhibits plasticity throughout the lifespan and adopts adaptive strategies as compensatory responses following injury or neurological disorder. However, examining these changes is challenging when relying solely on superficial qualitative assessments and, more importantly, in the absence of a guiding theoretical framework. Over the past decade or two, several hypotheses have been proposed to explain these adaptive mechanisms, including dynamic interaction theory and the concepts of motor redundancy (Singh et al., 2018b) and motor abundance (Latash, 2010, 2012), which relate to modifications in kinematic and kinetic degrees of freedom. When integrated with appropriate experimental designs and analytical methods, these theoretical perspectives can provide deeper insights into adaptive and developmental changes in human movement. Our previous studies have incorporated such theoretical, experimental, and analytical approaches to examine adaptive strategies (Singh et al., 2018a,b, 2020, 2023). Nevertheless, a substantial gap remains in the literature regarding the characterization of adaptive and developmental neuromuscular changes during human motion. Therefore, this Research Topic assembles six original research articles and one brief research report (*) that highlight recent trends and findings on neuromuscular plasticity during pathological and developmental movement. Below is the summary of submissions made to our Research Topic, categorized based on injury, neurological disorder, and lifespan changes in neuromuscular control.

### Injury

Song et al. investigated differences in neural function among individuals with varying functional abilities several years after anterior cruciate ligament reconstruction (ACLR). Participants were classified into three groups: (1) copers (COPs), (2) non-copers (NP), and (3) healthy controls (HC). Resting-state functional magnetic resonance imaging (fMRI) was used to assess blood oxygen level–dependent (BOLD) activity across these groups. The study revealed a shift in brain activity from somatosensory cortical areas toward subcortical regions, including the cerebellum and basal ganglia, where regional homogeneity was higher in the COPs group. This enhanced subcortical synchronization suggests an efficient return to sport among COPs.

###  Neurological disorder

Four studies in this Research Topic address the complex adaptations seen in common neurological disorders, exploring conditions from stroke and cerebral palsy (CP) to Parkinson's disease. Komaris et al. performed muscle synergy analysis on stroke patients to determine the basis of motor impairment; they found that the muscle synergies' dimensions and composition are preserved, whereas the temporal activation coefficient varies, suggesting flexible recruitment of fixed motor patterns. Researchers in this study suggested that targeted intervention of these altered temporal activations could aid in stroke rehabilitation.

Chmara et al. found that intensive exoskeleton therapy in patients with CP improves gait efficiency but not its symmetry, spatiotemporal parameters, or deviation scores. However, Clewes et al.^*^ found that alterations in gait kinematics, which lead to abnormal gait patterns, correlate with the severity of neuromuscular impairment, as evaluated using the clinical Gait Evaluation with Neuromuscular Impairments (GENI) tool.

Finally, Rathke et al. introduced a novel perturbation-evoked potentials system (PES) to probe cortical responses during gait. Their pilot study revealed that individuals with Parkinson's disease exhibited significantly longer N1 latencies compared to healthy controls, suggesting delayed sensorimotor processing which may be attributed to cortical degradation.

###  Human development

Hou et al. showed transitional changes in ankle dynamics improvements in children from 3 to 4 years of development, whereas at 5 years of age, children exhibited refined multi-joint coordination and muscle output associated with stable and flexible gait patterns. Inns et al. found that older adults, compared to younger adults, exhibited significant differences in kinematic responses, such as longer double-support and shorter swing phases, regardless of perturbation. This suggests age-related differences in neural control. Moreover, muscle activation was higher in older adults, likely due to the increased demand on the plantar flexors and knee extensors.

## Conclusion

In summary, shown in Table 1, our Research Topic compiles original research articles and a brief report that present neuromuscular and neuromechanical adaptations as compensatory responses to injury and neurological disorders. The plasticity in human motor behavior is presented through changes in neuromuscular and neuromechanical function associated with sensorimotor development. Additionally, our Research Topic provides a novel implementation of tools, such as GENI and PES for clinical assessment.

**Table 1:** Summaries of Research Topics featured in neuromuscular and kinematic dynamics in human movement adaptation.

**Category**	**Authors**	**Key findings/contributions**
Injury	Song et al.	Found a shift in brain activity from somatosensory to subcortical regions (cerebellum and basal ganglia) in post-ACL reconstruction participants. Copers showed higher regional homogeneity, indicating efficient neural adaptation and return to sport.
Neurological disorder	Komaris et al.	Muscle synergy structure was preserved in stroke patients, but temporal activation coefficients varied, suggesting flexible recruitment of fixed motor patterns and potential targets for rehabilitation.
	Chmara et al.	Intensive exoskeleton therapy improved gait efficiency in cerebral palsy but did not affect symmetry, spatiotemporal parameters and gait deviation scores.
	Clewes et al. ^*^	Altered gait kinematics correlated with severity of neuromuscular impairment, supporting the clinical utility of the GENI assessment tool.
	Rathke et al.	Parkinson's patients exhibited longer and delayed N1 latencies with PES system, suggesting delayed cortical sensorimotor processing.
Human development	Hou et al.	Children aged 3–5 years demonstrated developmental transitions in ankle dynamics and refinement in multi-joint coordination by the age of five.
	Inns et al.	Older adults displayed longer double-support and shorter swing phases than younger adults, along with higher muscle activation, indicating age-related differences in neural control.

Our research focuses on lower limbs and gait rehabilitation. However, the tools, methods, and experimental designs used in this area can also be applied to studying neuromuscular and neuromechanical responses during upper limb injuries, upper limb neurological disorders, and their development across the lifespan. This opens the door for future investigations in upper limb motor rehabilitation. Although our research has employed cutting-edge technologies and methods, such as 3D motion analysis, EMG, fMRI, and EEG, simulation-based approaches to examine neuromuscular and neuromechanical aspects also represent promising directions for future studies.
